# Mutant p53—a potential player in shaping the tumor–stroma crosstalk

**DOI:** 10.1093/jmcb/mjz071

**Published:** 2019-08-20

**Authors:** Yan Stein, Ronit Aloni-Grinstein, Varda Rotter

**Affiliations:** 1 Department of Molecular Cell Biology, Weizmann Institute of Science, Rehovot 7610001, Israel; 2 Department of Biochemistry and Molecular Genetics, Israel Institute for Biological Research, Box 19, Ness Ziona 7410001, Israel

## Abstract

A plethora of studies suggest that the non-transformed cellular and non-cellular components of the tumor, collectively known as the tumor microenvironment, have a significant impact on the tumorigenic process. It was suggested that the microenvironment, which initially restricts tumor development, is recruited by the tumor and maintains a crosstalk that further promotes cancer progression. Indeed, many of the molecules that participate in the tumor**–**stroma crosstalk have been characterized. However, the crucial factors that are responsible for the initiation of this crosstalk or the ‘recruitment’ process remain poorly understood. We propose that oncogenes themselves may influence the ‘recruitment’ of the stromal cells, while focusing on mutant p53. Apart from losing its tumor-suppressing properties, mutant p53 gains novel oncogenic functions, a phenomenon dubbed mutant p53 gain of function (GOF). Here, we discuss possible ways in which mutant p53 may modulate the microenvironment in order to promote tumorigenesis. We thus propose that mutant p53 may serve as a key player in the modulation of the tumor**–**stroma crosstalk in a way that benefits the tumor. Further elucidation of these ‘recruitment’ processes, dictated by mutant p53, may be utilized for tailoring personalized therapeutic approaches for patients with tumors that harbor p53 mutation.

A plethora of studies suggest that the non-transformed cellular and non-cellular components of the tumor, collectively known as the tumor microenvironment, have a significant impact on the tumorigenic process. It was suggested that the microenvironment, which initially restricts tumor development, is recruited by the tumor and maintains a crosstalk that further promotes cancer progression. Indeed, many of the molecules that participate in the tumor**–**stroma crosstalk have been characterized. However, the crucial factors that are responsible for the initiation of this crosstalk or the ‘recruitment’ process remain poorly understood. We propose that oncogenes themselves may influence the ‘recruitment’ of the stromal cells, while focusing on mutant p53. Apart from losing its tumor-suppressing properties, mutant p53 gains novel oncogenic functions, a phenomenon dubbed mutant p53 gain of function (GOF). Here, we discuss possible ways in which mutant p53 may modulate the microenvironment in order to promote tumorigenesis. We thus propose that mutant p53 may serve as a key player in the modulation of the tumor**–**stroma crosstalk in a way that benefits the tumor. Further elucidation of these ‘recruitment’ processes, dictated by mutant p53, may be utilized for tailoring personalized therapeutic approaches for patients with tumors that harbor p53 mutation.

It is now well accepted that cancer development does not depend solely on cancer cell-autonomous characteristics. Ample evidence suggests that various normal cellular and non-cellular components found adjacent to the tumor, known as the tumor microenvironment or tumor stroma, play a major modulating role in different stages of tumor development (Hanahan and Weinberg, 2011; Quail and Joyce, 2013). The tumor microenvironment consists, among others, of a unique population of fibroblasts known as cancer-associated fibroblasts (CAFs), blood vessels, immune cells and mesenchymal stem cells. As opposed to cancer cells, the cells found in the tumor microenvironment are usually considered to be genetically stable (Quail and Joyce, 2013). Yet, they might harbor mutations in some specific cases, such as mesenchymal cells that are derived from cancer cells, which underwent a process of epithelial–mesenchymal transition, or in the rare cases of hereditary cancers, in which all the cells, including stromal cells, possess a germ-line mutation.

It was suggested that initially, the microenvironment may serve as a barrier to tumor formation, even in the presence of potentially oncogenic genetic aberrations in precancerous cells (Bissell and Hines, 2011). However, as the tumorigenic process progresses, the microenvironment may become permissive of tumor development and actively promote tumorigenesis (Bissell and Hines, 2011). Though the tumor–stroma interaction has been widely studied, the mechanisms accounting for the shift between tumor-attenuating and tumor-promoting phenotypes of the microenvironment are not entirely understood. Indeed, there have been extensive studies that characterized the crosstalk between the tumor and the microenvironment, identifying a wide variety of secreted molecules that affect the ‘recruitment’ of the microenvironment by the cancer cells (Hanahan and Coussens, 2012). However, the factors that initiate this process remain to be determined. Plausible candidates that may initiate the crosstalk between the tumor and the microenvironment are oncogenes, which may regulate the expression of genes and secreted factors that act in a non-cell autonomous manner. Mutant p53 is the most commonly mutated gene in cancer (Kandoth et al., 2013), and aside of losing its tumor suppressor activity, it also acquires inherent oncogenic functions, a phenomenon termed mutant p53 GOF (Brosh and Rotter, 2009). Thus, we suggest that it may be reasonable to consider mutant p53 as a potential regulator of factors that may affect stromal cells in a non-cell autonomous manner.

**Figure 1:**
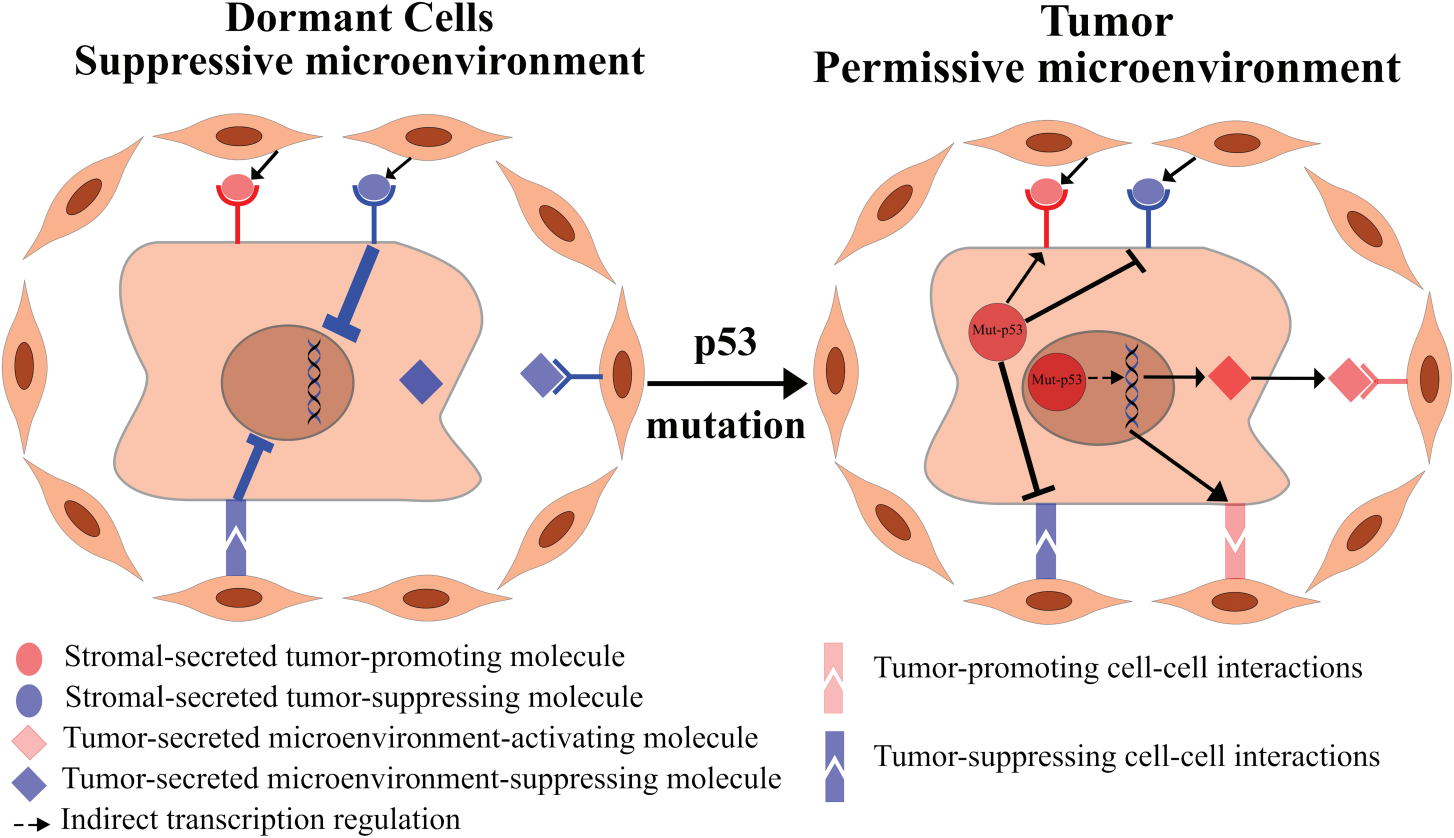
Mutant p53 modulates the tumor–stroma crosstalk to recruit the microenvironment to the benefit of the tumor. Dormant transformed cells are restricted by the microenvironment since the tumor-suppressing cues overcome the tumor-promoting cues, manifested by dormancy and tumor suppression (left panel). Mutant p53 in malignant tumor cells may lead indirectly to the transcription of secreted and cell–cell contact-dependent signaling molecules, which further instigates the secretion of pro-tumorigenic molecules by the stromal cells. In addition, mutant p53 might attenuate tumor-suppressing signals from the stroma, while enhancing tumor-promoting signals, thus shifting the balance toward a malignant, pro-tumorigenic state (right panel).

Though wild-type (WT) p53 has been extensively studied, most studies focused on its cell-autonomous functions. However, accumulating evidence suggests that p53 has a non-cell autonomous tumor-suppressing role, particularly in the tumor stroma (Bar et al., 2010). One of the modes by which WT p53 elicits its tumor-suppressing activity is by regulating the expression of various secreted proteins. For instance, p53 activation in the stromal cells induces the expression and secretion of various growth inhibitory molecules (Buckbinder et al., 1995; Komarova et al., 1998). In addition, p53 also suppresses the expression of tumor-promoting molecules. For example, it was demonstrated that SDF1 is negatively regulated by WT p53, as stromal p53 ablation contributed to the enhanced tumorigenic capacity of tumor cells, in an SDF1-dependent manner (Addadi et al., 2010). Intriguingly, fibroblasts harboring mutant p53 further augmented SDF1 expression compared to p53-null fibroblasts, which is also correlated with further enhanced tumor growth and metastasis formation upon co-injection with tumor cells to mice (Addadi et al., 2010). Thus, these findings not only indicate that WT p53 has a tumor-suppressing role in the tumor stroma, but also that mutant p53 has non-cell autonomous GOF properties, which may promote tumor growth. Although these findings suggest that p53 might have an important tumor-suppressive role in the tumor stroma, the relevance of stromal p53 mutations in human cancer is still controversial (Bar et al., 2010). A recent study demonstrated another non-cell autonomous tumor-suppressing activity of p53 *in vivo* (Lujambio et al., 2013). Conditional knockout of p53 in hepatic stellate cells enhanced liver fibrosis and cirrhosis, as well as tumorigenesis of the neighboring epithelial cells. This effects were mediated by ablation of the senescence-associated secretory phenotype and the conversion of macrophages to a pro-tumorigenic state (Lujambio et al., 2013). Our recent study indicates yet other non-cell autonomous functions of WT p53, pertaining to liver physiology. In this study, we demonstrated an interesting crosstalk between liver cells and lung cancer cells, which eventually leads to lung cancer cell migration, in a hepatic p53-dependent manner (Charni-Natan et al., 2018).

Though the aforementioned studies are consistent with the role of WT p53 as a tumor suppressor, a recent paper described a confounding finding, in which WT p53 seems to promote tumor-promoting functions of CAFs, suggesting that WT p53 may be implicated in the ‘education’ of stromal cells in the microenvironment (Arandkar et al., 2018). Another report by the same group demonstrated how tumor cells may suppress the activation of WT p53 in adjacent stromal cells, particularly in CAFs (Bar et al., 2009). Taken together, it is intriguing to speculate that p53 in stromal cells may be subject to ‘re-education’, first by attenuating its activation by tumor cells, then by transforming it into a GOF, ‘mutant’-like p53, in order to promote tumor growth in a non-cell autonomous manner. The exact mechanism by which this ‘re-education’ of p53 in the stromal compartment occurs remains to be elucidated.

While the non-cell autonomous role of WT p53 in stromal cells has been investigated, only few studies addressed how mutant p53, which is found predominantly in tumor cells, as well as being the most commonly mutated gene in cancer (Kandoth et al., 2013), affects the tumor–stroma crosstalk. In theory, mutant p53 may affect the tumor–stroma crosstalk in two different ways (Figure 1). First, a mutant p53-expressing tumor may induce the secretion of pro-tumorigenic factors, which lead to the recruitment of the microenvironment to support the cancer cells. Second, mutant p53 may modulate the signal of stromal-secreted molecules in a way that may benefit the tumor. We will discuss below these possibilities in detail and present evidence supporting each of them.

As mutant p53 is known to regulate transcription via interacting with other transcription factors (Brosh and Rotter, 2009; Muller and Vousden, 2013), it is reasonable to speculate that mutant p53 may affect the expression of various secreted proteins. Indeed, in our previous studies, we identified a secretory signature associated with mutant p53 and KRAS-harboring cancer cells, which we dubbed the cancer-related gene signature (CGS) (Buganim et al., 2010; Solomon et al., 2012). This CGS consisted of CXC chemokines such as CXCL1, pro-inflammatory interleukins such as IL-1β and IL-6, and extracellular matrix (ECM)-related proteins, such as the ECM remodeler, matrix metalloproteinase 3. Intriguingly, we found that different p53 mutations promoted the CGS in different underlying mechanisms. While p53 DNA contact mutations, namely p53^R248Q^ and p53^R273H^, promoted the CGS via nuclear factor κB (NF-κB) activation, p53 conformational mutants, namely p53^R175H^ and p53^H179R^, promoted the CGS by inhibiting BTG2, thus alleviating its inhibition on mutant H-Ras (Solomon et al., 2012). Another group reported a mutant p53-dependent upregulation of CXC chemokines as well, which was accompanied by increased, NF-κB and mutant p53-dependent, cell migration (Yeudall et al., 2012). Other papers showed that secreted molecules that are regulated by mutant p53 have pro-inflammatory effects. For instance, mutant p53 was shown to repress the expression of the secreted IL-1 receptor antagonist, therefore enhancing the responsiveness of mutant p53-expressing cells, and possibly the responsiveness of adjacent cells in the microenvironment, to IL-1 (Ubertini et al., 2015). Mutant p53 was also shown to alter the levels of various exosomal microRNAs, among which is miR-1246, which was upregulated in the exosomes of mutant p53-harboring cells (Cooks et al., 2018). In turn, these exosomes were taken up by adjacent macrophages, which caused their reprogramming to a pro-tumorigenic state, both *in vitro* and *in vivo*, in a mutant p53 and miR-1246-dependent manner (Cooks et al., 2018). An additional study that examined the transcriptional profile induced by mutant p53-overexpression on a p53-null background identified enrichment of secreted and transmembrane proteins among mutant p53 target genes, which implied a role of mutant p53 in the regulation of the secretome (Neilsen et al., 2011). Interestingly, this study reported an induction of invasiveness in naïve, non-mutant p53-expressing cancer cells upon administration of conditioned medium (CM) derived from mutant p53 overexpression cells, but not upon administration of CM derived from p53-null cells (Neilsen et al., 2011). This study may suggest an interesting mechanism, in which in a heterogeneous tumor comprised of mutant p53 and non-mutant p53-expressing cancer cells, the mutant p53-expressing cells can promote the invasiveness of all cancer cells, and perhaps also cells in the microenvironment, in a non-cell autonomous manner.

Mutant p53 was also shown to modulate the signal of various secreted molecules in a way that benefits cancer cells. For instance, it was reported that mutant p53 that is co-expressed with WT p53 in human bronchial epithelial cells inhibits the response of the cancer cells to transforming growth factor β1 (TGF-β1), thus suggesting a possible dominant-negative effect of mutant p53 over WT p53 (Gerwin et al., 1992). However, we were able to show that overexpression of mutant p53 on a p53-null background is able to attenuate the response to TGF-β1 via downregulating the expression of its receptor, TGF-βR2, thus demonstrating a true mutant p53 GOF (Kalo et al., 2007). In our recent experiments, we identified a mechanism in which mutant p53 in cancer cells may enhance the signal of secreted molecules from the stroma in order to promote drug resistance (unpublished data). While different studies demonstrated the phenomenon of stromal-mediated drug resistance (Straussman et al., 2012; McMillin et al., 2013) and mutant p53 cell-autonomous mechanisms for drug resistance (Shetzer et al., 2014), to the best of our knowledge, the cooperative effect of mutant p53 and stromal-derived molecules on drug resistance has not been demonstrated yet. Another cancer-associated process in which mutant p53 is implicated is chronic inflammation, which is a well-accepted hallmark of cancer (Hanahan and Weinberg, 2011) and a contributor to cancer progression (Coussens and Werb, 2002). Indeed, numerous studies showed that the tumor microenvironment has a significant impact on the perpetuation of the chronic inflammatory state, thus contributing to tumor progression (de Visser and Coussens, 2006). In our study, we were able to show that mutant p53 is able to enhance the activation of NF-κB by TNFα, while concomitantly suppressing the pro-apoptotic effect of TNFα (Weisz et al., 2007). A following study corroborated these results in a mouse model of colorectal cancer (Cooks et al., 2013). Mice heterozygous for mutant p53 were more susceptible to chronic inflammation induced by dextran sodium sulfate than mice heterozygous for a knockout p53 allele, thus rendering them prone to the development of colon carcinoma. Similar results were obtained in mice that were homozygous for mutant p53 allele compared to p53 homozygous knockout mice, thus demonstrating a true mutant p53 GOF (Cooks et al., 2013). Yet another study confirmed these results in breast cancer cells and demonstrated that this effect is mediated by a protein–protein interaction between mutant p53 and the DAB2IP (Di Minin et al., 2014). This interaction shifted the activation pattern of downstream TNFα effectors, causing an enhanced NF-κB activation as well as decreased ASK1/JNK1 activation, which leads to increased invasiveness of the cancer cells (Di Minin et al., 2014). In that regard, it is worth noting that while p53 mutations are considered to occur at a late stage in sporadic colorectal cancer, mutant p53 is frequently detected early in colitis-associated colorectal cancer, and considered to be among the earliest mutations in this type of cancer (Ullman and Itzkowitz, 2011). Considering these results, it is tempting to speculate that mutant p53 may be a major contributor to the initiation of colitis-associated colorectal cancer, and perhaps in other types of cancers as well, by augmenting the initial inflammatory response into a chronic inflammation, which further contributes to carcinogenesis.

Finally, in our previous study, we were able to demonstrate an intricate crosstalk between patient-derived CAFs and lung cancer cells, in which mutant p53 induces the secretion of a factor from the neighboring cells and modulates the response to this factor in the cancer cells (Madar et al., 2013). We utilized a co-culture system in which we were able to characterize separately the transcriptional changes in CAFs, as well as in their co-cultured cancer cell counterparts, by separating them using fluorescence-activated cell sorting according to a distinct fluorescent label for the CAFs and the cancer cells. By comparing co-cultures of CAFs with cancer cells that were either p53-null or harboring mutant p53 alleles, we were able to identify a mutant p53-dependent signature in the CAFs, indicating an interferon-β (IFN-β) response elicited by the CAFs and triggered non-cell autonomously by the presence of mutant p53 in the cancer cells. Interestingly, the activation of the IFN-β response seemed to be dependent on cell–cell contact, since CM derived from mutant p53-harboring cells and CAFs caused increased activation of this response, while CM derived from tumor cells alone produced a much milder effect. In turn, mutant p53 caused an attenuation of the IFN-β activation in the cancer cells themselves by upregulating SOCS1, a negative regulator of the IFN pathway, therefore alleviating a suppressing effect of IFN-β on tumor cell migration. Thus, we demonstrated both non-cell autonomous function of cancer cell-expressed mutant p53, by inducing the IFN-β response in adjacent stromal cells, as well as modulation of the IFN-β signal by mutant p53, in a way that benefits the tumor cells.

In light of the aforementioned studies, it is tempting to speculate that mutant p53 in cancer cells may indeed serve as a potential stromal modulator. Mutant p53, as well as other oncogenes, may serve as both the initiators and the propagators of the tumor–stroma vicious cycle, during which they promote the ‘re-education’ of the cells in the stromal compartment, as well as causing a modulation of the signals arriving from the stromal cells in a way that benefits the tumor cells.

*[Varda Rotter is the incumbent of the Norman and Helen Asher Professorial Chair for Cancer Research at the Weizmann Institute. Yan Stein was supported by the Eshkol fellowship issued by the Israeli Ministry of Science, Technology and Space. Research in the laboratory of Varda Rotter is supported by a Center of Excellence Grant from the Israel Science Foundation (ISF) and a Center of Excellence Grant from the Flight Attendant Medical Research Institute (FAMRI).]*
